# Simulation at Mach 2 flow of ethylene/air reacting mixture within a cavity flame holder

**DOI:** 10.1016/j.heliyon.2024.e24961

**Published:** 2024-01-23

**Authors:** Zachary Chapman, David Peterson, Jeffrey Doom

**Affiliations:** a South Dakota State University, United States of America; b Air Force Research Laboratory, United States of America

## Abstract

The ongoing simulation of flame holder cavities remains a pivotal aspect in the advancement of scramjet engine development. This study aims to evaluate the applicability of Reynolds Averaged Navier-Stokes (RANS) turbulence models in simulating supersonic reacting flows within flame holder cavities. RANS remains the standard approach for engineering simulations in this regime, so it is important to understand how different RANS models perform. Four RANS turbulence models, the k-*ϵ*, Realizable k-*ϵ*, k-*ω* SST, and $\bar{v^{2}}$-*f* models, are used for the simulation of a flame holder cavity at Mach 2 using two different chemical mechanisms. Results were compared to experimental data and prior simulation results from the Air Force Research Laboratory (AFRL). The $\bar{v^{2}}$-*f* turbulence model was found to provide the best overall results and often provided similar or superior results to previous results using a higher fidelity hybrid RANS/LES approach. Additionally, the two chemical mechanisms are compared, with the smaller of the two mechanisms being found to provide better results when used with the RANS models investigated in this work.

## 1 Introduction

In aerospace engineering, the pursuit of achieving hypersonic speeds has become a focal point of technological advancement. Hypersonic flight, characterized by velocities exceeding Mach 5, presents many challenges, one of which is the development of efficient and reliable propulsion systems. Among the critical components of hypersonic propulsion, flameholders play a pivotal role in ensuring stable combustion in the high-speed airflow. Hypersonic propulsion systems are designed to propel vehicles through the Earth's atmosphere at speeds that surpass five times the speed of sound. Achieving and sustaining such velocities demands innovative engineering solutions to address the unique aerodynamic and thermal challenges associated with hypersonic flight. At the heart of these propulsion systems, flameholders serve as crucial elements in scramjets, a promising form of a hypersonic propulsion system, that enables sustained combustion within the engine. The flame holder cavity is central in managing combustion under extreme conditions. In the hypersonic regime, where airflows are supersonic, maintaining stable and efficient combustion becomes a formidable task. The flameholder cavity is designed to stabilize the flame, ensuring that combustion remains continuous and controlled.

The investigation into flameholder cavities has attracted considerable attention in the realm of combustion research. Notable studies, such as those conducted by Wang et al. [3], have leveraged Large Eddy Simulation (LES) techniques in conjunction with a passive scalar method to numerically model combustion processes. Wang et al. work involve comparisons with $H_{2}$ jet simulations in a simplified cavity geometry, which demonstrates the promise of the passive scalar mixing in comprehending combustion phenomena.

Peterson et al. [2] expanded on this investigation by conducting simulations on a supersonic non-reacting flame holder. They utilized both Reynolds-Averaged Navier-Stokes (RANS) and a hybrid RANS-LES model. While their results were compared against experimental data, with the hybrid model showing improved agreement, challenges persisted in certain regions. Ruan et al. [4] delved into the capabilities of LES in predicting compressible flows in multi-species reacting scenarios, particularly within a cavity-based scramjet. Their findings highlighted the success of the LES model in capturing accurate kinetics, albeit with some reduction, emphasizing combustion occurrences within the largely subsonic mixing layer above the cavity.

Qi et al. [6] directed their attention to optimizing fuel injection parameters in supersonic scramjets. By conducting simulations with different injection angles, they observed the impact on shock wave angle, penetration height, and span expansion area of kerosene fuel droplets. Kummitha [7] explored passive scalar techniques for enhancing air and fuel mixture in scramjet engines, evaluating the efficacy of small bumps at the lower wall, uniform zigzag surfaces, and parabola-shaped cavities of the combustion chamber. The study's findings indicated that parabola cavities and bumps with a parabola shape served as crucial flame holders. Meanwhile, the uniform zigzag surface technique demonstrated enhanced mixing and combustion efficiency.

Cao et al. [8] contributed insights into fuel injection schemes using ethylene, comparing cases where all ethylene is injected upstream versus a combination of upstream and cavity floor injection. Their observations emphasized the importance of injection strategies in achieving optimal combustion efficiency within the cavity region.

Generally, research on flame-holder cavities falls into two categories: techniques for enhancing flame-holding capabilities and advancements in modeling techniques. This paper aims to investigate the applicability of RANS turbulence models for simulating combustion within a flame holder cavity. In contrast to computationally intensive methods such as Hybrid RANS-LES or LES simulations, RANS turbulence models offer a more computationally efficient alternative. The paper compares three commonly used RANS turbulence models and introduces the $\bar{v^{2}}$-*f* RANS model, offering a distinct approach to modeling the boundary layer. Additionally, two chemical mechanisms, Baurle [12] and Fuerby [13], are compared for their impact on the simulation results. The subsequent sections delve into the governing equations, numerical methods, finite-rate chemistry, geometry, meshing methods, and turbulence models implemented in OpenFOAM. The results and conclusions are discussed in Sections 7 and 8, respectively, providing a comprehensive exploration of the intricate interplay between RANS turbulence models and combustion dynamics within flame holder cavities.

## 2 Governing equations

The governing equations are the reacting, compressible, Unsteady, Navier–Stokes equations (URANS). The equations are the Conservation of Mass, Equation (1); Species, Equation (2); Momentum, Equation (3); and Energy, Equation (4).

(1) $$\frac{\partial \bar{\rho }}{\partial t}+\frac{\partial \bar{\rho }{\overset{˜}{u}}_{j}}{\partial x_{j}}=0,$$

(2) $$\frac{\partial \bar{\rho }{\overset{˜}{Y}}_{n}}{\partial t}+\frac{\partial \bar{\rho }{\overset{˜}{Y}}_{n}{\overset{˜}{u}}_{j}}{\partial x_{j}}=\frac{\partial }{\partial x_{j}}\left[\left(\bar{\rho }D_{k}+\frac{\mu_{t}}{Sc_{t}}\right)\frac{\partial {\overset{˜}{Y}}_{n}}{\partial x_{j}}\right]+{\overset{˙}{\omega }}_{n},$$

(3) $$\frac{\partial \bar{\rho }{\overset{˜}{u}}_{i}}{\partial t}+\frac{\partial \bar{\rho }{\overset{˜}{u}}_{i}{\overset{˜}{u}}_{j}}{\partial x_{j}}=-\frac{\partial \bar{p}}{\partial x_{i}}+\frac{\partial }{\partial x_{j}}\left[{\bar{\tau }}_{ij}-\bar{\rho }\overset{˜}{u_{i}^{\prime }u_{j}^{\prime }}\right]$$

(4) $$\frac{\partial \bar{\rho }\overset{˜}{h}}{\partial t}+\frac{\partial \bar{\rho }\overset{˜}{h}{\overset{˜}{u}}_{j}}{\partial x_{j}}=\frac{\partial }{\partial x_{j}}\left[\left(\bar{\rho }\alpha +\frac{\mu_{t}}{Pr_{t}}\right)\frac{\partial \overset{˜}{h}}{\partial x_{j}}\right]+{\overset{˙}{\omega }}_{h}$$

In these equations $\bar{\rho }$, ${\overset{˜}{Y}}_{k}$, ${\overset{˜}{u}}_{j}$, and $\overset{˜}{h}$ are mean quantities for density, chemical species n, velocity, and enthalpy. $\frac{}{\tau_{ij}}$ is the stress tensor for viscosity, and *α* is the thermal diffusivity. In these equations, $Pr_{t}$ is the turbulent Pandtl number which has a value of 0.9 and $Sc_{t}$ is the turbulent Schmidt number, also having a value of 0.9. ${\overset{˙}{\omega }}_{n}$ is the reaction rate for species n and ${\overset{˙}{\omega }}_{h}$ is the source term for combustion.

The equation of state is given by the ideal gas law and the Reynolds stress is modeled as shown by Equation (5).

(5) $$-\bar{\rho }\overset{˜}{u_{i}^{\prime }u_{j}^{\prime }}=\mu_{t}\left(\frac{\partial {\overset{˜}{u}}_{i}}{\partial x_{j}}+\frac{\partial {\overset{˜}{u}}_{j}}{\partial x_{i}}-\frac{2}{3}\frac{\partial {\overset{˜}{u}}_{k}}{\partial x_{k}}\delta_{ij}\right)+\frac{2}{3}\bar{\rho }k$$

## 3 Numerical methods

Simulating supersonic reacting flows poses a substantial challenge due to the extensive computational resources needed. To tackle this issue, the open-source solver OpenFOAM was selected for the simulations, cited as [9], owing to its capacity for running massively parallel simulations. OpenFOAM offers two solvers for supersonic flow: rhoCentralFOAM, a density-based solver, and sonicFOAM, a pressure-based solver specifically designed for supersonic flow. Our solver is a combination of sonicFOAM and reactingFOAM. ReactingFOAM is a transient solver for simulating compressible reacting flows which uses CHEMKIN [10] format for finite rate chemistry. All numerical details are covered by the OpenFOAM website [9].

## 4 Finite rate chemistry

Concerning finite rate chemistry, we examine a chemical system, denoted as (6), comprising *N* species involved in *M* reactions as described by Poinsot and Veynante [11]:

(6) $$\sum_{j=1}^{N}\nu_{kj}^{\prime }\mu_{k}⇌\sum_{j=1}^{M}\nu_{kj}^{\prime \prime }\mu_{k}$$

for j=1,M where $\mu_{k}$ is a symbol for species *k*. $\nu_{kj}^{\prime }$ and $\nu_{kj}^{\prime \prime }$ are the stoichiometric coefficients of species *k* for *j* reactions. The reaction term (7), (8) is defined as:

(7) $${\overset{˙}{\omega }}_{k}=W_{k}\sum_{j=1}^{M}\nu_{kj}{\overset{ˆ}{Q}}_{j}$$

where

(8) $${\overset{ˆ}{Q}}_{j}=\underset{\mathrm{forward reaction}}{\underset{︸}{K_{ij}\prod_{k=1}^{N}{\left(\frac{\rho Y_{k}}{W_{k}}\right)}^{\nu_{kj}^{\prime }}}}-\underset{\mathrm{reverse reaction}}{\underset{︸}{K_{rj}\prod_{k=1}^{N}{\left(\frac{\rho Y_{k}}{W_{k}}\right)}^{\nu_{kj}^{\prime \prime }}}}.$$

Using the empirical Arrhenius law (9),

(9) $$K_{ij}=A_{ij}T^{\beta_{j}}\exp\left(-\frac{E_{j}}{RT}\right)=A_{ij}T^{\beta_{j}}\exp\left(-\frac{T_{a_{j}}}{T}\right).$$

Therefore, the source term (10) is:

(10) $${\overset{˙}{\omega }}_{k}=W_{k}\sum_{j=1}^{M}\nu_{kj}\left[A_{ij}T^{\beta_{j}}\exp\left(-\frac{T_{a_{j}}}{T}\right)\prod_{k=1}^{N}{\left(\frac{\rho Y_{k}}{W_{k}}\right)}^{\nu_{kj}^{\prime }}\right]-W_{k}\sum_{j=1}^{M}\nu_{kj}\left[A_{rj}T^{\beta_{j}}\exp\left(-\frac{T_{a_{j}}}{T}\right)\prod_{k=1}^{N}{\left(\frac{\rho Y_{k}}{W_{k}}\right)}^{\nu_{kj}^{\prime \prime }}\right].$$

The chemical mechanisms used in this project are Bauele et al. [12], which has 7 species and 3 reactions, and the Fuerby et al. [13], which has 23 species and 6 reactions.

Baurle mechanism is shown in Table 1. The first column is the chemical reactions. The second column is the preexponential constant ($A_{ij}$). The third column is the temperature exponent ($\beta_{j}$) and the fourth column is the activation energy ($E_{j}$).

**Table 1:** Baurle mechanism.

Reaction	*A*	*b*	*E*_*j*_
*C*_2_*H*_4_ + *O*_2_⇔2*CO* + 2*H*_2_	2.23E+11	0.21	15858.0
2*CO* + *O*_2_⇔2*CO*_2_	3.64E+08	1.86	9874.0
2*H*_2_ + *O*_2_⇔2*H*_2_	2.55E+17	−0.77	3.6

## 5 Geometry and mesh

**Figure 1:**
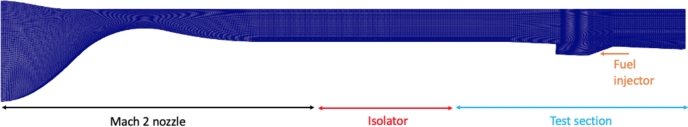
Grid used for simulations.

A supersonic combustion chamber consisting of a nozzle, an isolator, and the test section, including a flame holder cavity, made up the geometry that was simulated (Fig. 1). The height of the constant area isolator upstream of the cavity is 5.08 cm, and the cavity height is 1.65 cm. Detailed dimensions are provided in [5]. The experimental cavity is equipped with 11 ethylene ($C_{2}H_{4}$) fuel injectors evenly spaced. A small width, 1.27 cm, of the combustion chamber was simulated using cyclic boundary conditions to reduce the size of the mesh. This width included one fuel injector. The experimental study had a freestream total pressure of 483 kPa and a total temperature of 589 with a total flow rate of 56 SLPM of fuel injected with 11 equally spaced fuel injectors. For our simulations, a total pressure of 483 kPa and a total temperature of 589 K was used for the inlet boundary condition to provide Mach 2 at the isolator. For the fuel injector, velocity inlet boundary conditions were used where the velocity was set to 27.5 m/s at standard pressure and temperature which matches the total flow rate of 56 SLPM with 11 injectors for ethylene. The simulation conditions are listed in Table 2.

**Table 2:** Simulation conditions.

Parameter	Air	Fuel
*p*_0_ [kPa]	483	–
*T*_0_ [K]	589	–
$Y_{O_{2}}$	0.233	0
$Y_{N_{2}}$	0.767	0
$Y_{C_{2}H_{4}}$	0	1

Meshing was done using OpenFOAM's meshing tool blockMesh. To generate the mesh, the geometry is split into several blocks by defining the endpoints of each block. The number of cells is then specified for each block in the *x*, *y*, and *z* directions. BlockMesh generates meshes of hexahedral cells. The mesh used for the simulations is shown in Fig. 1. The mesh sensitivity tests were done and the mesh of 3.5 million cells is adequate for our simulations of supersonic cavity flame holder [14]. The mesh used had a maximum y+ of approximately 55 and a skewness angle of approximately 20.

The fuel was ignited by adding a heat source within the cavity region. The heat source was left on until the fuel was ignited and turned off. The heat source acted similar to a “spark plug”. The spark was turned off at 0.0025 seconds. The simulation ran for a total time of 0.02 seconds. Statistics were turned on for our unsteady simulation from a time of 0.005 seconds to 0.02 seconds. Fureby mechanism compared to the Baurle mechanism was around 5 times greater to simulate.

## 6 Mesh sensitivity

**Figure 2:**
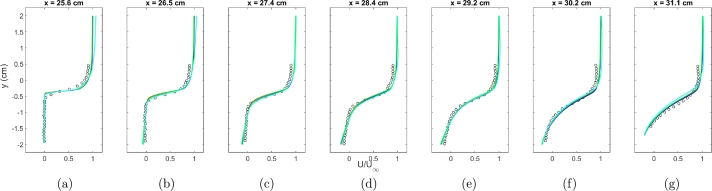
Normalized component of *U* velocity for experimental data (symbols), 1.5 million cells (cyan), 3.5 million cells (green), 5 million cells (blue), and 10 million cells (red).

**Figure 3:**
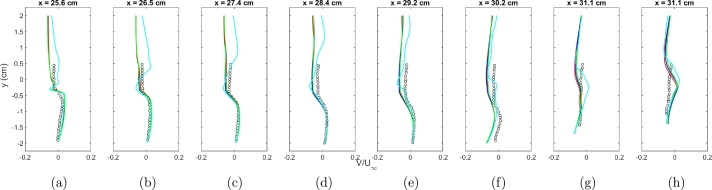
Normalized component of *V* velocity for experimental data (symbols), 1.5 million cells (cyan), 3.5 million cells (green), 5 million cells (blue), and 10 million cells (red).

The outcomes of the mesh sensitivity test for the RANS turbulence model are illustrated in Figs. 2a-g and 3a-g. The tests involved grid sizes of 1.5 million cells, 3.5 million cells, 5 million cells, and 10 million cells. Analyzing the results of the RANS mesh sensitivity test, it is observed that all meshes yield similar results for the U component of velocity. Discrepancies emerge when examining the V component of velocity. The 1.5 million cell grid exhibits the least favorable results as anticipated, with no substantial improvement noted beyond the 3.5 million cell grid.

## 7 Turbulence modeling

A comparison was conducted among four RANS turbulence models, namely, Standard k-*ϵ* [16], k-*ω* SST [17], Realizable k-*ϵ* [15], and $\bar{v^{2}}$-*f* [18]. RANS turbulence models were preferred over DES and LES turbulence models due to their lower sensitivity to boundary layer modeling, unlike LES and DES turbulence models. The equations defining these turbulence models in OpenFOAM [9] are presented below. It is noteworthy that, in this study, no adjustments were made to the constants in the RANS turbulence models.

### 7.1 Standard k-*ϵ*

The k-*ϵ* turbulence model, introduced by Launder [16], is a two-equation turbulence model widely employed for various problems. The turbulent kinetic energy and turbulent kinetic energy dissipation rates are expressed by Equations (11) and (12), respectively, with the turbulent viscosity defined by Equation (13).

(11) $$\frac{D}{Dt}\left(\rho k\right)=\nabla \cdot \left(\rho D_{k}\nabla k\right)+P-\rho \epsilon$$

(12) $$\frac{D}{Dt}\left(\rho \epsilon \right)=\nabla \cdot \left(\rho D_{\epsilon }\nabla \epsilon \right)+\frac{C_{1}\epsilon }{k}\left(P+C_{3}\frac{2}{3}k\nabla \cdot u\right)-C_{2}\rho \frac{\epsilon^{2}}{k}$$

(13) $$\nu_{t}=C_{\mu }\frac{k^{2}}{\epsilon }$$

### 7.2 k-*ω* SST

The k-*ω* SST turbulence model, an enhanced variant of the standard k-*ω* model introduced by Menter [17], is a two-equation model. The equation governing the turbulent kinetic energy is given by Equation (14), while the equation for the turbulent specific dissipation rate is defined by Equation (15). The turbulence viscosity is determined by Equation (16).

(14) $$\frac{D}{Dt}\left(\rho k\right)=\nabla \cdot \left(\rho D_{k}\nabla k\right)+\rho G-\frac{2}{3}\rho k\left(\nabla \cdot u\right)-\rho \beta^{⁎}\omega k+S_{k}$$

(15) $$\frac{D}{Dt}\left(\rho \omega \right)=\nabla \cdot \left(\rho D_{\omega }\nabla \omega \right)+\frac{\rho \gamma G}{\nu }-\frac{2}{3}\rho \gamma \omega \left(\nabla \cdot u\right)-\rho \beta \omega^{2}-\rho \left(F_{1}-1\right)CD_{k\omega }+S_{\omega }$$

(16) $$\nu_{t}=a_{1}\frac{k}{\max\left(a_{1}\omega ,b_{1}F_{23}S\right)}$$

### 7.3 Realizable k-*ϵ*

The Realizable k-*ϵ* model, introduced by Shih [15], represents a modification of the standard k-*ϵ* model. This variant has demonstrated reliability in various applications, including rotating homogeneous shear flows and boundary-free shear flows with mixing layers, among others [15]. It is a two-equation turbulence model, with the turbulent kinetic energy defined by Equation (17) and the turbulent dissipation defined by Equation (18). The turbulence viscosity is determined by Equation (19), where $C_{\mu }$ is defined by Equation (20).

(17) $$\frac{D}{Dt}\left(\rho k\right)=\nabla \cdot \left(\rho D_{k}\nabla k\right)+\rho G-\frac{2}{3}\rho \left(\nabla \cdot u\right)k-\rho \epsilon +S_{k}$$

(18) $$\frac{D}{Dt}\left(\rho \epsilon \right)=\nabla \cdot \left(\rho D_{\epsilon }\nabla \epsilon \right)+C_{1}\rho |S|\epsilon -C_{2}\rho \frac{\epsilon^{2}}{k+{\left(\nu \epsilon \right)}^{0.5}}+S_{\epsilon }$$

(19) $$\nu_{t}=C_{\mu }\frac{k^{2}}{\epsilon }$$

(20) $$C_{\mu }=\frac{1}{A_{0}+A_{s}U^{⁎\frac{k}{\epsilon }}}$$

### 7.4 $\bar{v^{2}}$-*f*

The $\bar{v^{2}}$-*f* turbulence model, as proposed by Durbin [18], is a four-equation turbulence model that has demonstrated improved performance in applications such as jet impingement heat transfer [19] and other heat transfer problems [20]. Despite its success in these areas, it appears that this turbulence model has not been widely considered for simulating supersonic cavities. The turbulent kinetic energy and dissipation rate are defined by Equations (21) and (22), respectively. Additionally, the model incorporates a relaxation function defined by Equation (23). The turbulence stresses normal to the streamlines are determined by Equation (24), where *α* is defined by Equation (25).

(21) $$\frac{D}{Dt}\left(\rho k\right)=\nabla \cdot \left(\rho D_{k}\nabla k\right)+\rho G-\frac{2}{3}\rho \left(\nabla \cdot u\right)k-\rho \epsilon +S_{k}$$

(22) $$\frac{D}{Dt}\left(\rho \epsilon \right)=-\nabla \cdot \left(\rho u\epsilon \right)+\nabla \cdot \left(\rho D_{\epsilon }\nabla \epsilon \right)+C_{\epsilon 1}\rho \frac{G}{T_{s}}-\frac{2}{3}C_{\epsilon 1}+C_{\epsilon 3}\rho \left(\nabla \cdot u\right)\epsilon -C_{\epsilon 2}\frac{\rho }{T_{s}}\epsilon +S_{\epsilon }$$

(23) $$-\nabla \cdot^{2}f=-\frac{f}{L^{2}}-\frac{1}{L^{2}k}\left(\alpha -C_{2}G\right)$$

(24) $$\frac{D}{Dt}\left(\rho v^{2}\right)=\nabla \cdot \left(\rho D_{k}\nabla v^{2}\right)+\rho \min\left(kf,C_{2}G-\alpha \right)-N\rho \frac{\epsilon }{k}v^{2}+S_{v^{2}}$$

(25) $$\alpha =\frac{1}{T_{s}}\left(\left(C_{1}-N\right)v^{2}-\frac{2}{3}k\left(C_{1}-1\right)\right)$$

## 8 Results

The objective of this study was to assess the use of RANS turbulence models to simulate ethylene combustion in a supersonic flame holder cavity at Mach 2. Two different chemical mechanisms, Baurle (7 species, 3 reactions) [12] and Fuerby (23 species, 66 reactions) [13], are also used for simulation and are compared.

### 8.1 Baurle

**Figure 4:**
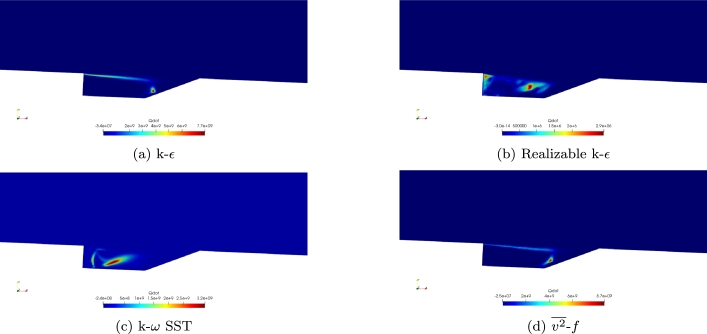
Comparison of heat release for the four turbulence models using the Baurle mechanism.

Simulations were first conducted with the Baurle mechanism with each of the four turbulence models. The heat release for each of the four turbulence models is shown in Fig. 4a-d. Comparing the four turbulence models, the k-*ϵ* and $\bar{v^{2}}$-*f* provide similar values and distributions. Both show a small region with the maximum heat release where ignition is occurring. Both show a higher region near the top of the cavity where heat transfer is occurring between the cavity and the free stream. The SST model provides lower values and does not provide a similar distribution. The Realizable k-*ϵ* model also provides a much more uneven distribution and much lower values.

**Figure 5:**
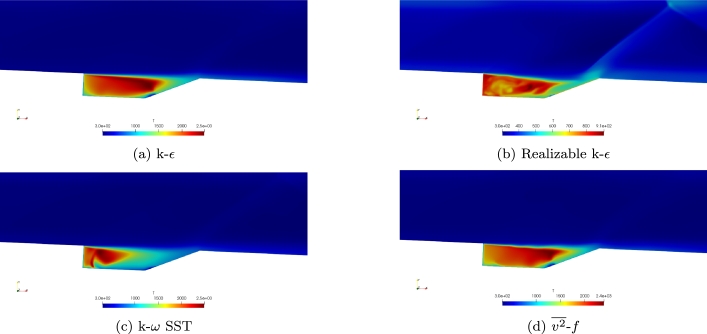
Comparison of temperature for the four turbulence models using the Baurle mechanism.

The temperature distribution for each of the turbulence models is shown in Fig. 5a-d. Again, the k-*ϵ* and $\bar{v^{2}}$-*f* turbulence models show excellent levels of agreement. The SST turbulence model provides similar maximum temperatures but does not provide a similar distribution. The Realizable k-*ϵ* model results in much lower values of temperature and appears to cause no ignited.

**Figure 6:**
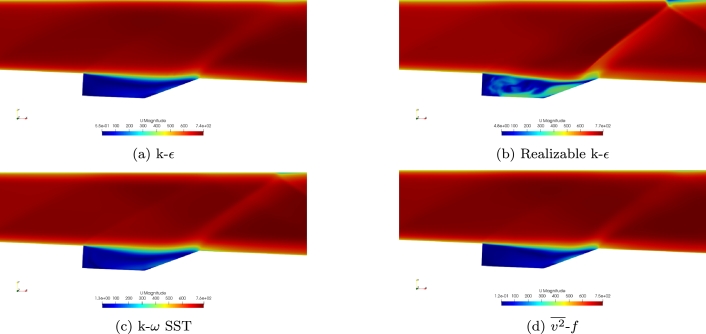
Comparison of velocity magnitude for the four turbulence models using the Baurle mechanism.

The velocity magnitude for the four turbulence models is shown in Fig. 6a-d. Each of the four turbulence models provides similar results within the free stream. Each of the turbulence models shows a free stream velocity of approximately Mach 2 which demonstrates that the inflow conditions were captured accurately. The Realizable k-*ϵ* model does show differences within the cavity and shows higher velocities within the cavity, unlike the other four turbulence models.

**Figure 7:**
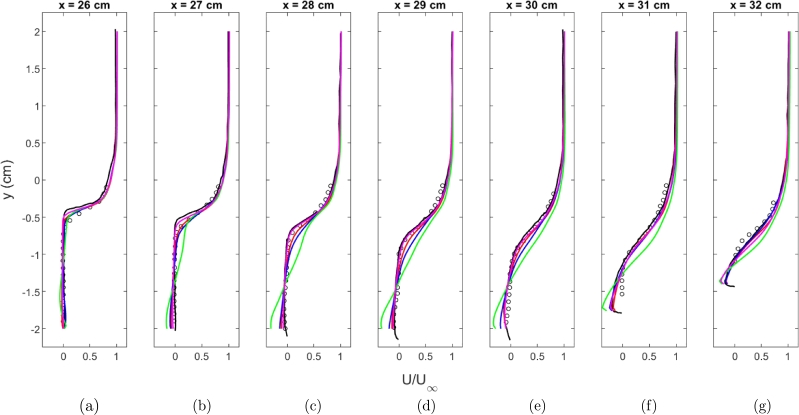
U component velocity profiles using the Baurle mechanism. Experimental data from [1] ∘, Simulation results from [1] k-*ϵ* Realizable k-*ϵ* k-*ω* SST $\bar{v^{2}}$-*f*

Velocity profiles were extracted at seven locations within the cavity and were plotted. The results are normalized with the free stream velocity. Also, plotted are experimental results and simulation results from the results in Peterson and Hassan [1]. The profiles are shown in Fig. 7a-g. To provide a more quantitative analysis, the error between each experimental data point in the profiles of 7 and the closest computational cell is calculated. The error does not necessarily provide a direct indication of the level of accuracy of the simulation but is instead used to compare how well each of the different models approximates the experiment. Table 3 shows the average error for each of the four turbulence models at each profile location, as seen in Fig. 7 and also provides an average overall profile location. Except for the Realizable k-*ϵ* turbulence model, each of the turbulence models can capture the velocity profiles. The Realizable k-*ϵ* turbulence model appears to have a problem capturing the shear layer and over-predicts the velocities within the shear layer. Examining Fig. 7 and Table 3, the $\bar{v^{2}}$-*f* turbulence model provides the best agreement with the experimental data. The k-*ϵ* model provides the second best fit closely followed k-*ω* SST model. The k-*ϵ* model seems to provide a slight under-prediction of the velocity while the $\bar{v^{2}}$-*f* model provides a slight over-prediction.

**Table 3:** Comparison of average error for normalized U component of velocity for the Baurle mechanism.

Location [cm]	k-*ϵ*	Realizable k-*ϵ*	k-*ω* SST	$\bar{v^{2}}$-*f*
26.0	0.0287	0.0381	0.0269	0.0283
27.0	0.0357	0.0812	0.0402	0.0311
28.0	0.0422	0.1270	0.0524	0.0283
29.0	0.0453	0.1293	0.0607	0.0304
30.0	0.0446	0.1281	0.0647	0.0289
31.0	0.0566	0.1256	0.0609	0.0563
32.0	0.0656	0.1316	0.0444	0.0799
Average	0.0455	0.1087	0.0500	0.0405

**Figure 8:**
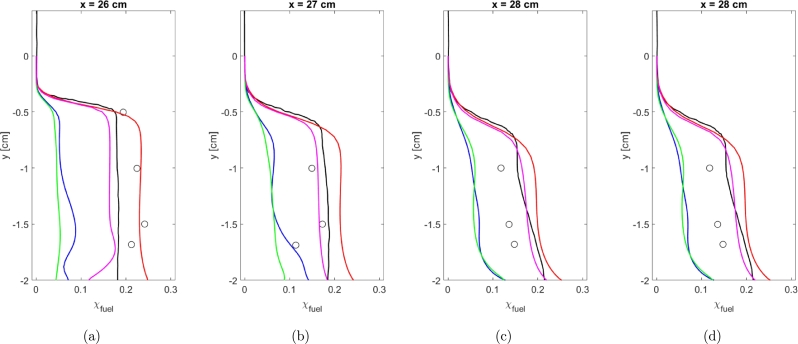
Fuel concentrations using Baurle Mechanism. Experimental data from [1] ∘, Simulation results from [1] k-*ϵ* Realizable k-*ϵ* k-*ω* SST $\bar{v^{2}}$-*f*

Mixing data was collected experimentally using a laser-based technique which provides a molecular measurement of fuel concentration that cannot tell the difference between burnt and unburnt fuel [2]. Mixing data is plotted in Fig. 8a-d. Mixing data is calculated using Equation (26).

(26) $$\chi_{fuel}=1-\frac{\chi_{N_{2}}}{\chi_{N_{2}}^{\infty }}$$

Results for each of the turbulence models are plotted and compared to experimental data and previous simulation data from the AFRL. It should be noted that results were collected with a consistent turbulent Schmidt number. The value of the turbulent Schmidt number was not optimized for any of the models. The k-*ω* SST and Realizable k-*ϵ* turbulence models severely under-predict the fuel concentrations at all locations within the cavity. Both the k-*ϵ* and $\bar{v^{2}}$-*f* models provide good agreement with the experimental data and previous simulation results from the AFRL. The k-*ϵ* model does appear to provide over-predictions of fuel concentration at all locations while the $\bar{v^{2}}$-*f* turbulence model seems to initially under-predict and then over-predict fuel concentration. At locations further downstream of the cavity, the k-*ϵ* and $\bar{v^{2}}$-*f* turbulence models do provide closer agreement with each other.

Table 4 provides the average error for mixing data in the same fashion as Table 3. As seen in Fig. 8, the Realizable k-*ϵ* and k-*ω* SST models provide poor fits to the data. The $\bar{v^{2}}$-*f* model does again provide a better fit to the experimental data compared to the k-*ϵ* model and provides a level of agreement similar to the much more advanced Hybrid RANS/LES simulations of the [2].

**Table 4:** Comparison of average error for mixing data for the Baurle mechanism.

Location [cm]	k-*ϵ*	Realizable k-*ϵ*	k-*ω* SST	$\bar{v^{2}}$-*f*
26.0	0.0142	0.1711	0.1506	0.0551
27.0	0.0692	0.0819	0.0664	0.0252
28.0	0.0652	0.0728	0.0699	0.0416
29.0	0.0767	0.0765	0.0747	0.0781
Average	0.0563	0.1006	0.0904	0.0500

### 8.2 Fuerby

**Figure 9:**
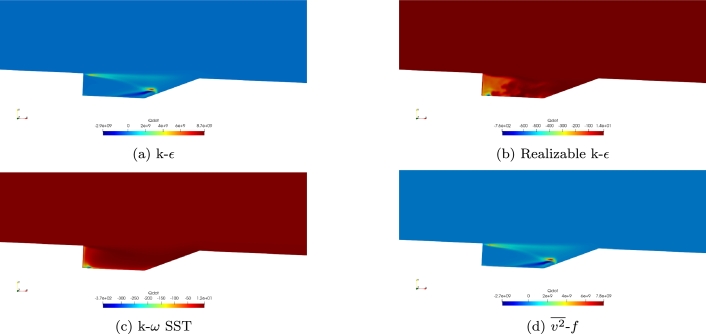
Comparison of heat release for the four turbulence models using the Fuerby mechanism.

The Fuerby mechanism was then used for simulations at Mach 2. Shown in Fig. 9a-d is heat released during combustion. Similar to the simulations conducted with the Baurle mechanism, the k-*ϵ* and $\bar{v^{2}}$-*f* turbulence models compare well to each other. Both show similarities in contours. The maximum values are both found to be in the same range and the ignition of fuel does seem to occur in the same location. Compared to the results using the Baurle mechanism, similar maximum values of heat release are found. Both the Realizable k-*ϵ* and k-*ω* SST models are unable to stay ignited with the Fuerby mechanism as indicated by the low levels of heat release.

**Figure 10:**
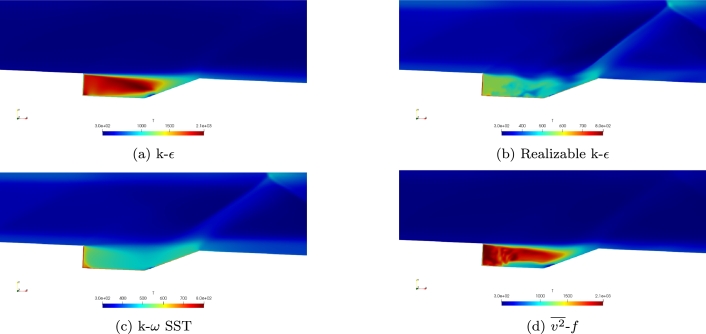
Comparison of temperature for the four turbulence models using the Fuerby mechanism.

Temperature contours of the Mach 2 simulations using the Fuerby mechanism are shown in Fig. 10a-d. As shown with the heat release, Fig. 9b-c, the Realizable k-*ϵ* and k-*ω* SST models do not stay ignited when the Fuerby mechanism is used for simulation. This can be seen from the low temperatures within the cavity. Comparing the temperature contours of the k-*ϵ* and $\bar{v^{2}}$-*f* turbulence models, the $\bar{v^{2}}$-*f* model appears to have a less uniform temperature distribution than the k-*ϵ* turbulence model. The $\bar{v^{2}}$-*f* turbulence model seems to be more unsteady than the k-*ϵ* model when the Fuerby mechanism is used. Compared to the maximum temperatures found from the simulations using the Baurle mechanism, the Fuerby mechanism does find lower temperatures.

**Figure 11:**
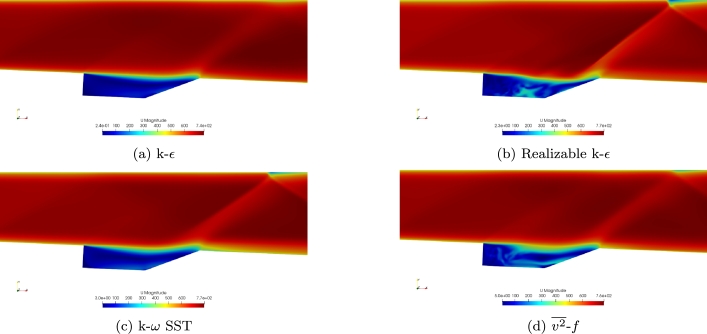
Comparison of velocity magnitude for the four turbulence models using the Fuerby mechanism.

Contours of velocity magnitude for the Mach 2 simulations using the Fuerby mechanism are shown in Fig. 11a-d. Similar results are found using the Fuerby mechanism as the Baurle mechanism. At the experimental conditions investigated, combustion does not greatly change the velocity field within the cavity. This is also seen in the experimental data. Each of the turbulence models was able to capture the free stream velocities. The Realizable k-*ϵ* turbulence model does result in higher velocities within the cavity. Unlike simulations with the Baurle mechanism, the $\bar{v^{2}}$-*f* turbulence model also results in higher velocities within the cavity similar to the Realizable k-*ϵ* turbulence model. This again seems to be due to the $\bar{v^{2}}$-*f* model becoming more unsteady when the Fuerby mechanism is used.

**Figure 12:**
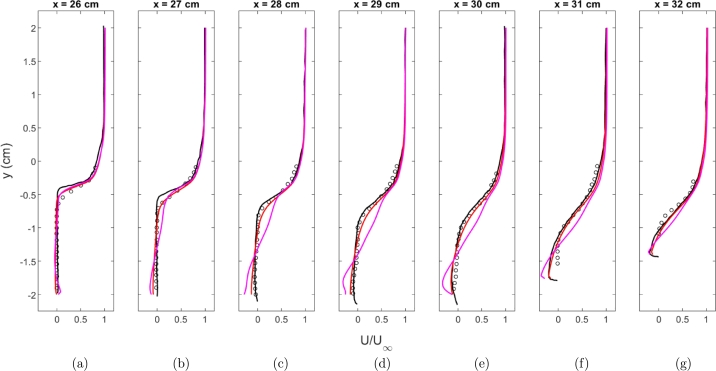
U component velocity profiles using the Fuerby mechanism. Experimental data from [1] ∘, Simulation results from [1] k-*ϵ*$\bar{v^{2}}$-*f*

Velocity profiles at several locations were extracted at the same locations within the cavity. These profiles are shown in Fig. 12a-g. Profiles for the k-*ω* SST and Realizable k-*ϵ* turbulence model are not plotted because the models did not stay ignited during the simulations. The k-*ϵ* turbulence model does seem to provide a better agreement to the velocity profiles than the $\bar{v^{2}}$-*f* turbulence model when the Fuerby mechanism is used. The $\bar{v^{2}}$-*f* model provides a good agreement to the experimental data near the start of the cavity but has issues capturing the shear layer closer to the injector similar to the Realizable k-*ϵ* model when the Baurle mechanism is used. Table 5 shows the average error for the k-*ϵ* and $\bar{v^{2}}$-*f* models at each location the velocity profiles were extracted. As expected, the $\bar{v^{2}}$-*f* model provides worse agreement compared to the k-*ϵ* model.

**Table 5:** Comparison of average error for normalized U component of velocity for the Fuerby mechanism.

Location [cm]	k-*ϵ*	$\bar{v^{2}}$-*f*
26.0	0.0314	0.0320
27.0	0.0374	0.0664
28.0	0.0475	0.1123
29.0	0.0510	0.1267
30.0	0.0480	0.1159
31.0	0.0532	0.1042
32.0	0.0560	0.0941
Average	0.0464	0.0931

**Figure 13:**
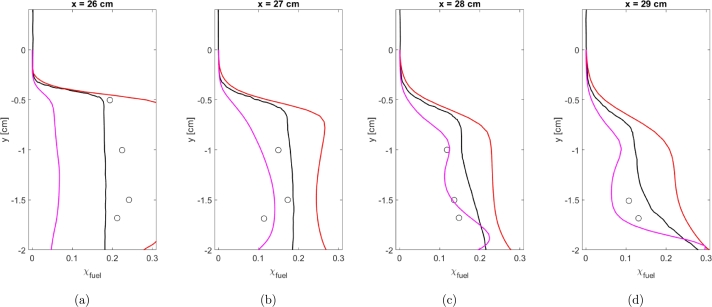
Fuel concentrations using Baurle Mechanism. Experimental data from [1] ∘, Simulation results from [1] k-*ϵ* Realizable k-*ϵ* k-*ω* SST $\bar{v^{2}}$-*f*

Mixing data was again calculated using Equation (26) and plotted. Again, results are only included for the k-*ϵ* and $\bar{v^{2}}$-*f* turbulence models. The average error is shown in Table 6. Unlike the velocity profiles, the $\bar{v^{2}}$-*f* model does provide better agreement to the mixing data compared to the k-*ϵ* turbulence model. As seen in Fig. 13a-d, at the location furthest from the injector the k-*ϵ* model provides a severe over-prediction of the mixing data while the $\bar{v^{2}}$-*f* model under-predicts the mixing data. Closer to the injector, the $\bar{v^{2}}$-*f* model does provide a closer level of agreement to the mixing data and appears to provide a similar or superior level of agreement than the results from [2].

**Table 6:** Comparison of average error for mixing data for the Fuerby mechanism.

Location [cm]	k-*ϵ*	$\bar{v^{2}}$-*f*
26.0	0.0945	0.1597
27.0	0.1047	0.0346
28.0	0.1011	0.0135
29.0	0.1196	0.0427
Average	0.1050	0.0626

### 8.3 Comparison of Baurle and Fuerby mechanisms

Table 7, Table 8 show a comparison of the average error between the Baurle and Fuerby mechanisms for the velocity and mixing profiles respectively. Results are only shown for the k-*ϵ* and $\bar{v^{2}}$-*f* turbulence models since the Realizable k-*ϵ* and k-*ω* SST turbulence models were not considered for the Fuerby mechanism. When comparing the velocity the Baurle mechanism does give the best agreement to the experimental data when using the $\bar{v^{2}}$-*f* model which provides the best agreement overall. Minimal differences are seen between the average error for the k-*ϵ* model for both mechanisms. Interestingly, the Baurle mechanism seems to provide better results at locations further from the injector while the Fuerby mechanism does better near the injector.

**Table 7:** Comparison of average error of velocity profiles between Baurle and Fuerby mechanisms.

Location [cm]	Baurle		Fuerby	
	k-*ϵ*	$\bar{v^{2}}$-*f*	k-*ϵ*	$\bar{v^{2}}$-*f*
26.0	0.0287	0.0283	0.0314	0.0320
27.0	0.0357	0.0311	0.0374	0.0664
28.0	0.0357	0.0283	0.0475	0.1123
29.0	0.0422	0.0304	0.0510	0.1267
30.0	0.0453	0.0289	0.0480	0.1159
31.0	0.0566	0.0563	0.0532	0.1042
32.0	0.0656	0.0799	0.0560	0.0941
Average	0.0455	0.0405	0.0454	0.0931

**Table 8:** Comparison of average error of mixing profiles between Baurle and Fuerby mechanisms.

Location [cm]	Baurle		Fuerby	
	k-*ϵ*	$\bar{v^{2}}$-*f*	k-*ϵ*	$\bar{v^{2}}$-*f*
26.0	0.0142	0.0551	0.0945	0.1597
27.0	0.0692	0.0252	0.1047	0.0346
28.0	0.0652	0.0416	0.1011	0.0135
29.0	0.0767	0.0781	0.1196	0.0427
Average	0.0563	0.0500	0.1050	0.0626

When considering the mixing data, again the $\bar{v^{2}}$-*f* model using the Baurle mechanism gives the best overall agreement. The k-*ϵ* turbulence model using the Baurle mechanism does give better results than either turbulence model using the Fuerby mechanism as well. Comparing the $\bar{v^{2}}$-*f* results between the Baurle and Fuerby mechanisms, it is again seen that the Baurle mechanism appears to perform better further from the injector while the opposite is true for the Fuerby mechanism. This could be because the Baurle mechanism is over-predicting the fuel concentration which corrects further away from the injector while the Fuerby mechanism under-predicts fuel concentration which is exaggerated further from the injector.

## 9 Summary

The simulation of supersonic flame holder cavities remains important for the development of scramjet engines however simulations of scramjet engines, and affordable simulations, particularly for reacting flows, are needed to make engineering decisions. This work seeks to assess the use of different RANS turbulence models for the simulation of reacting supersonic flows in flame holder cavities. In this work, the k-*ϵ*, Realizable k-*ϵ*, k-*ω* SST, and $\bar{v^{2}}$-*f* turbulence models are assessed for the simulation of reacting flows in supersonic flame holder cavities using a custom OpenFOAM solver, rssFOAM. Results are compared to experimental data on velocity and mixing profiles within the cavity and past simulation results using hybrid RANS/LES turbulence modeling. Additionally, simulations were conducted with two different chemical mechanisms. Results show that the k-*ϵ* and $\bar{v^{2}}$-*f* turbulence models consistently give the highest levels of agreement to the experimental data. To the author's knowledge, this is the first application of the $\bar{v^{2}}$-*f* model for flows in this regime at Mach 2, and the results suggest that further investigation of this model should be considered. Additionally, comparing the chemical mechanisms, differences are seen in the level of agreement when the same turbulence model is used for both velocity and mixing profiles. This is due to the coupling between the combustion and the velocity field. One surprising result of this work is the performance of the SST model. The authors are currently unable to give a definitive answer as to the poor performance of SST however ongoing work using a commercial code has also seen poor performance. One possible explanation could be the coefficient of the SST model is not tuned to adequately capture the physics of this problem, however, due to the high number of variables, this is hard to pin down and more work would be required to determine the cause of the poor performance. Overall, the $\bar{v^{2}}$-*f* turbulence model simulated with the Baurle mechanism gave the overall best results for both velocity and mixing profiles. This combination results in the lowest error when compared to the experimental data, however, the Baurle mechanism does give better results with either turbulence model. This indicates that the Baurle mechanism does a better job of modeling this problem.

## Disclaimer

The views expressed are those of the authors and do not reflect the official guidance or position of the United States Government, the Department of Defense, or the United States Air Force. Distribution is unlimited; AFRL-2023-1509, cleared 31 March 2023.

## CRediT authorship contribution statement

**Zachary Chapman:** Writing – review & editing, Writing – original draft, Visualization, Validation, Software. **David Peterson:** Writing – review & editing, Writing – original draft, Visualization, Validation, Software. **Jeffrey Doom:** Writing – review & editing, Writing – original draft, Visualization, Validation, Software.

## Declaration of Competing Interest

The authors declare that they have no known competing financial interests or personal relationships that could have appeared to influence the work reported in this paper.

## Data Availability

Data available upon request.
